# Pharmacokinetics of drospirenone and ethinylestradiol in Caucasian and Japanese women

**DOI:** 10.3109/13625187.2012.677076

**Published:** 2012-06-10

**Authors:** Hartmut Blode, Kristin Kowal, Katrin Roth, Stefanie Reif

**Affiliations:** * Bayer HealthCare Global R&D Centre, Beijing, China; † Bayer HealthCare Pharmaceuticals, Berlin, Germany

**Keywords:** Drospirenone, Ethinylestradiol, Pharmacokinetics, Ethnic origin

## Abstract

**Objective** To investigate the pharmacokinetics of drospirenone (DRSP) and ethinylestradiol (EE) in Caucasian and Japanese women.

**Method** Three open-label, non-randomised studies were performed to assess the pharmacokinetics following single doses of EE 0.02 mg/DRSP 3 mg or DRSP monotherapy (1, 3 or 6 mg) in Caucasian (Study 1) and Japanese (Study 2) women, and daily doses with EE 0.02 mg/DRSP 3 mg over 21 consecutive days in Caucasian and Japanese women (Study 3).

**Results** In Studies 1 and 2, there was a linear dose-dependent increase in DRSP C_max_ and systemic exposure across the range of doses used in both ethnic groups. The coadministration of EE had no relevant effect on the pharmacokinetic parameters of 3 mg DRSP. In Study 3, steady-state DRSP concentrations were achieved after about eight days of treatment in both ethnic groups with approximately a threefold accumulation.There was about a twofold EE accumulation over 21 days in both ethnic groups. There were no differences in DRSP or EE exposure at day 21 between ethnic groups; the ratio of the geometric means (Japanese/Caucasian) of the AUC_0−24h_ were 1.05 (90% CI: 0.95–1.17) and 1.02 (90% CI: 0.76–1.38), respectively.

**Conclusion** Ethnic origin had no clinically relevant influence on the pharmacokinetics of DRSP and EE.

## INTRODUCTION

Ethinylestradiol (EE) and drospirenone (DRSP) are the active components of several oral contraceptives (OCs). The standard dose is EE 0.03 mg/DRSP 3 mg (e.g., Ocella™, Barr Laboratories, Inc.; Syeda™, Sandoz; Yasmin®, Bayer HealthCare Pharmaceuticals; Zarah®, Watson Pharmaceuticals) and a low-dose EE/DRSP formulation has also been developed consisting of EE 0.02 mg within betacyclodextrin (betadex) clathrate in combination with DRSP 3 mg (EE 0.02 mg/DRSP 3 mg, e.g., Gianvi™, Teva Pharmaceuticals; Loryna™, Sandoz; Yaz®, Bayer HealthCare Pharmaceuticals). The inclusion of EE within a betadex-clathrate when combined with DRSP (as in EE 0.02 mg/DRSP 3 mg) does not affect single-dose pharmacokinetics and the relative bioavailability of either EE or DRSP^1^.

The pharmacokinetic profile of multiple doses of EE 0.02 mg/DRSP 3 mg in different ethnic groups has not been assessed so far. Some early studies suggested that ethnic differences may alter the pharma-cokinetics of contraceptive steroids^2^–^4^, whereas others pointed to the absence of such an effect^5^,^6^. In one study however, pharmacokinetic differences following administration of a potential combined OC containing ORG30659 and EE were demonstrated between Japanese and Caucasian women^7^.

In the clinical development of the EE 0.02 mg/DRSP 3 mg OCs, three studies were performed in Caucasian and Japanese women with the aim of comparing the pharmacokinetic profile between the two ethnic groups. These studies included pharmacokinetic analysis of single and multiple doses of 0.02 mg/DRSP 3 mg as well as single doses of DRSP (1, 3, and 6 mg). The results of these studies are reported here.

## METHODS

### Study design

Studies 1 and 2 (single-dose studies) were both non-randomised, open-label, dose-escalation studies with identical design elements. Study 1 took place at a single centre in Germany between November 2000 and April 2001, and included only Caucasian women. Study 2 was also conducted at a single centre, in this case in Japan, between December 2000 and June 2001; it included only Japanese women. Study 3 (multiple-dose study) was an open-label, non-randomised, parallel-group comparison with respect to ethnic origin which involved two centres between August and December 2001; it included Caucasian and Japanese women in Germany and Japan, respectively.

All three studies complied with national regulations for good clinical practices, with the ethical principles of the Declaration of Helsinki (amended in 1996), and with the guidelines of the International Conference on Harmonisation. The studies were approved by the appropriate local ethics committees, and all participants gave written and informed consent before enrolment.

### Subjects

Healthy women aged 20–35 years were eligible to participate in all three studies. Participants were required to have a body mass index (BMI) of ≥ 18 and ≤ 26 kg/m^2^, at least three normal menstrual cycles following childbirth, abortion or lactation, and to be willing to use non-hormonal contraceptive methods (e.g., non-medicated intrauterine devices, condoms, diaphragms, spermicidal vaginal suppositories or abstinence) during treatment cycles.

The enroled women underwent a washout period of at least one cycle (28 days) if they had used OCs prior to the study, or of at least six months if they had used long-acting reversible contraceptives (injectables, implants or intrauterine systems) containing steroids. Use of antibiotics and medications that may induce or inhibit liver enzyme production during the eight weeks (Studies 1 and 2) or four weeks (Study 3) that preceded participation in the study was another cause for exclusion. Former smokers could be recruited provided they had stopped smoking at least three months before treatment began. Further exclusion criteria comprised: a history of relevant diseases (including complications during pregnancy [icterus, severe pruritus, cholestasis, herpes gestationis, otosclerosis and gestational diabetes], liver disorders, tumours, thromboembolic diseases or complications, metabolic or thyroid disorders), systolic blood pressure (BP) < 90 or > 140 mmHg, diastolic BP < 50 or > 90 mmHg, heart rate < 50 or > 100 beats/min, clinically relevant findings in the physical or gynaecological examination (including breasts), abnormal Pap smear, undiagnosed vaginal bleeding, blood or plasma donation within four weeks (Studies 1 and 2, and German women in Study 3) or 16 weeks (Japanese women in Study 3) of study commencement; concomitant medication use; diets incompatible with the standard study meals; and a positive pregnancy or urine drug test.

### Treatment

In Studies 1 and 2, 36 Caucasian women and an equal number of Japanese women, respectively, were assigned to one of four single dose treatments as follows: DRSP 1 mg (*n* = 6); DRSP 3 mg (*n* = 6); DRSP 6 mg (*n* = 6); and EE 0.02 mg/DRSP 3 mg (*n* = 18). The study drug was administered 3–5 days after the onset of menstruation by a member of the study team to ensure the participant received the treatment as planned and that this information was documented. The women fasted for at least ten hours prior to tablet intake and were permitted only standardised meals 2, 5, 8 and 11 hours after treatment. Drinking water was allowed except for one hour before and after study drug administration. Study drugs were taken with 200 mL of non-carbonated water at room temperature. The women were required to maintain an upright posture (sitting, standing or walking) for at least four hours after treatment. Twenty-four hours after treatment, they could resume their normal diet except for alcohol and grapefruit-containing food, which were prohibited 48 hours before and only permitted 168 hours after treatment.

Study 3 involved 24 Caucasian women and an equal number of Japanese women. Each received EE 0.02 mg/DRSP 3 mg once daily for 21 consecutive days. The first tablet was taken two to four days after the onset of menstruation. Drug administration took place either at the study site (under supervision of a member of the study team on days 1, 2, 5, 9, 12, 15, 18 and 21) or at home (self-administered on all other days). Participants were instructed to take the study drug at about the same time each day (± one hour) and to record the time of intake in diary cards. Compliance was assessed by analysing recordings in diary cards along with the number of tablets consumed. The women fasted for at least ten hours prior to tablet intake on pharmacokinetic profile days (days 1 and 21), on safety blood sampling days (days 2 and 9), and before the post-treatment safety examination (day 26, 27 or 28). Drinking water was allowed except for one hour before and after study drug administration. Study drugs were taken with 200 mL of non-carbonated water at room temperature. On the two pharmacokinetic profile days (days 1 and 21), women were required to maintain an upright posture (sitting, standing or walking) for at least four hours after treatment intake. Standardised meals were provided 2, 5, 8 and 11 hours after drug administration. The women were permitted their normal diet on other study days except for alcohol (not permitted one day before drug administration on days 1, 20 and 21) and grapefruit-containing food or beverage (not permitted for two days before first tablet intake and until seven days after last tablet intake).

### Pharmacokinetic assessments

In Studies 1 and 2, blood samples (7.5 mL for EE and/or 2.7 mL for DRSP analyses) were collected via an indwelling cannula into additive-free syringes before and 0.5, 1, 1.5, 2, 4, 6, 8, 10, 12, 16, 24, 34, 48, 72, 96, 120, 144 and 168 hours after drug intake (up to 72 hours for EE samples). In Study 3, on day 1 and day 21, blood samples (9 mL in total for EE and DRSP) were collected as described in Studies 1 and 2, before and 0.5, 1, 1.5, 2, 4, 6, 8, 10, 12 and 16 hours after study drug intake for EE and DRSP analyses. Additional blood samples were obtained 24, 34, 48 and 72 hours after the last tablet intake on day 21 for EE and DRSP analyses, and 96, 120, 144 and 168 hours after the last tablet intake on day 21 for additional DRSP analyses. Blood samples to determine trough levels of DRSP were taken just before tablet intake on days 2, 5, 9, 12, 15, 18 and 21. Serum was prepared from blood samples by centrifugation and stored between −18°C and −25°C until analysis.

The concentration of DRSP in serum was measured by a validated radioimmunoassay as described elsewhere^8^. The lower limit of quantification for DRSP was 0.25–0.5 ng/mL. Interassay coefficient of variation ranged between 5.2% and 11.2%, and mean accuracy between 92% and 108%. The concentration of EE in serum was measured by a validated gas chromatogra-phy/mass spectrometry (GC/MS), with a lower limit of quantification of 10 pg/mL. For determination, EE was extracted from serum samples with toluene. The samples were centrifuged, frozen and the organic phase was decanted into a clean silylated glass tube. The organic phase was then evaporated to dryness with nitrogen, derivatised with pentafluorobenzoyl chloride (PFBCI) and n-methyl-n-trimethyl silyl-trifluoroacetamide (MSTFA). An aliquot was injected into the GC/MS system. Sample separation was achieved with a gas chromatograph HP6890 (Hewlett-Packard) using a fused silica column (5% phenylmethylsilicone, HP-5) with a length of 10 – 12.5 m, an internal diameter of 0.20 mm and a film thickness of 0.33 μ m. Samples were analysed with a quadrupole mass selective detector 5973 (Hewlett-Packard) in negative chemical ionisation mode applying methane as reagent gas. The peak area of EE and the internal standard (deuterium-labelled EE) were measured. Interassay coefficient of variation for EE ranged between 9.1% and 11.7%, and mean accuracy between 96% and 101%.

In Study 3, sex hormone-binding globulin (SHBG) levels were determined at baseline and from the same pre-dose (trough) serum samples collected for DRSP assessment on days 5, 9, 12, 15, 18 and 21 prior to study drug administration, and in the serum samples collected subsequently after 96, 120, 144 and 169 hours following the last tablet intake on day 21. SHBG was measured in the serum samples using the SHBG IRMA CT-Q3 kit manufactured by Zentec, a commercially available, validated assay. The lower limit of quantification for SHBG was 16.8 nmol/L and the interassay coefficient of variation ranged between 5.6% and 10.3%.

### Safety and tolerability assessments

At screening in Studies 1 and 2 (single-treatment studies), information was gathered about demographic data, medical history (general and gynaecological), use of medication, smoking history, alcohol intake and diet; a physical examination (including height, body weight, blood pressure, and heart rate), a gynaecological examination (including a cervical smear), a pregnancy test on urine, and a urinalysis were carried out; and blood was drawn for the laboratory examinations. Urine drug screening was performed and samples for laboratory tests were collected 30 minutes before treatment. After two days and 5–14 days following drug administration the laboratory examinations were repeated. Fourteen to 26 days following drug administration the pregnancy test on urine and the urinalysis were repeated. Women were given the opportunity to report the occurrence of adverse events (AEs) during, and up to eight days after, treatment.

In Study 3, a general physical examination (including body weight and vital signs), a gynaecological examination, an electrocardiogram and clinical laboratory testing (blood chemistry, haematological examination and urinalysis) were carried out at screening. For safety assessment, all clinical laboratory tests were repeated on days 1, 2, 9, 21 (samples taken before administration of study drug) and 5 – 7 days after last tablet intake (i.e. on day 26, 27 or 28). The women were given the opportunity to report the occurrence of AEs during, and up to 14 days after, treatment.

In all three studies, all AEs were coded using the Hoechst Adverse Reactions Terminology System (HARTS; version 2.3), and classified by the study investigators with regard to their intensity (mild, moderate or severe) and to their likely relationship to study medication (i.e. none, unlikely, possible, probable or definite).

### Pharmacokinetic evaluation

The observed maximum serum drug concentration (C_max_) and time to reach this concentration (t_max_) were taken directly from individual concentration versus time data. The terminal half-life (t_½_) was determined from the terminal rate constant (λ_z_) which was calculated by means of regression analysis from the linear part of the concentration–time curve in a semi-logarithmic plot (resulting t_½_ calculated as t_½_ = In2/λ_z_). The area under the concentration–time curves (e.g., up to 24 hours after dosing [AUC_(0–24h)_] or up to the last measurable time point [AUC_(0–last)_]) for EE and DRSP was calculated using the linear trapezoidal rule. The AUC value for DRSP was calculated according to the following equation:

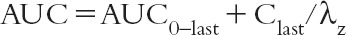


(with C_last_ as concentration at last data point).

The oral clearance (CL/F) was obtained for DRSP from the ratio of the dose (D) and the corresponding AUC value:

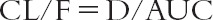


(where F is the absolute bioavailability).

The pharmacokinetic parameters of DRSP (C_max_, AUC_(t-last)_,AUC) were considered as the primary variables in Studies 1 and 2. All other pharmacokinetic parameters, including those of EE, were considered as secondary variables.

In Study 3, DRSP AUC_(0–24h)_ on day 21 was considered the primary variable; other pharmacokinetic parameters on day 21 and day 1 were secondary variables. In addition, the accumulation ratio (R_A_) was calculated from the ratio of the AUC_(0–24h)_ value for day 21 and day 1 according to:

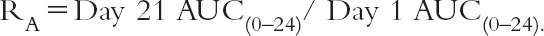


### Sample size and statistical analysis

Studies 1 and 2 were designed assuming that the pharmacokinetic data of DRSP after a single oral dose of EE 0.02 mg/DRSP 3 mg in Caucasian and Japanese women would be compared in a subsequent analysis. It was assumed that the primary variable was approximately log-normally distributed with common variances on the log-scale for both ethnic groups. Mean differences between Japanese and Caucasian volunteers in log-transformed variables were back-transformed to obtain ratios on the original scale along with 90% confidence intervals (CIs). Assuming (i) a coefficient of variation (CV) of 25% for the primary target variables, (ii) that the bioavailability ratio would lie between 0.91 and 1.10, and (iii) that at least 90% of the women would be included in the per-protocol set, 18 women receiving EE 0.02 mg/DRSP 3 mg would have to be recruited in each study in order to have a 90% probability that the 90% CI of the bio-availability ratio between Japanese and Caucasian women was within the range 0.70 to 1.43. No statistical comparisons were planned for the DRSP monotherapy treatments. In order to obtain descriptive data, a sample size of six volunteers for 1 mg, 3 mg and 6 mg DRSP treatments was chosen without statistical justification.

In Study 3, 24 women would have to be recruited in either ethnic group in order to have a probability of at least 90% so that the 90% CI of the AUC_(0–24h)_ ratio between Japanese and Caucasian women would be within the range of 0.70–1.43, taking similar assumptions into account as mentioned before, i.e. that the coefficient of variation was 25% for AUC_(0–24h)_, that the AUC_(0–24h)_ ratio would lie between 0.91 and 1.10, and that at least 70% of the women would be included in the per-protocol population.

All pharmacokinetic parameters were summarised using descriptive statistics separately for each drug dose and ethnic group. In Study 3, the AUC_(0–24h)_ of DRSP and EE on day 21 were log-transformed and the mean difference between Japanese and Caucasian women was calculated on the log-transformed values. The 90% CI for the difference on the log-scale was determined using a normal approximation. Mean differences and the confidence limits in log-transformed AUC_(0–24h)_ were back-transformed to obtain ratios on the original scale along with 90% CIs.

## RESULTS

### Subjects

In Study 1, of the 53 Caucasian women screened, 36 received the study drug and completed the study. In Study 2, of the 64 Japanese women screened, 36 received study drug and completed the study. In Study 3, of the 94 women screened, 48 started treatment (Caucasian *n* = 24; Japanese *n* = 24). With the exception of one Caucasian woman who discontinued treatment in Study 3, all volunteers completed the study as per protocol and are included in the pharmacokinetic analyses. The demographic characteristics of the women from all three studies are presented in Table 1. The ethnic groups did not differ with respect to baseline characteristics with the exception of height and body weight, which tended to be lower in Japanese compared with Caucasian women.

### Protocol deviations

In Study 1, there were 13 women who had at least one minor protocol deviation (procedure deviation: *n* = 3, mostly ‘tablet intake at home’; time schedule deviation: *n* = 10, mostly ‘blood sampling too early or too late’), one woman had two protocol deviations in Study 2 (blood and urine samples obtained on day 19 instead of between days 5 and 14) and, in Study 3, 11 women had at least one protocol deviation (inclusion criteria deviation: *n* = 1; time schedule deviation: *n* = 2; procedure deviations: *n* = 11, mostly ‘no fasting state for blood sampling’). All of these protocol deviations were considered to be minor and did not affect the pharmacokinetic evaluation.

**Table 1:** Summary characteristics of the study participants. Results are presented as mean (range).

	*Study 1*	*Study 2*	*Study 3*	
			
Parameter	*Caucasian (n = 36)*	*Japanese (n = 36)*	*Caucasian (n = 24)*	*Japanese (n = 24)*
Age (years)	28 (22−35)	29 (20−34)	28 (20−34)	27 (20−35)
Height (cm)	171 (156−182)	160 (151−174)	168.8 (154−188)	158.3 (149−168)
Body weight (kg)	62 (48−74)	53 (42−80)	62.9 (51−78.5)	53.3 (42−71)
Body mass index (kg/m^2^)	21.4 (18.1−25.4)	20.6 (18.0−26.4)	22.1 (18.0−26.0)	21.2 (18.0−26.0)

### Pharmacokinetics of drospirenone

**Figure 1:**
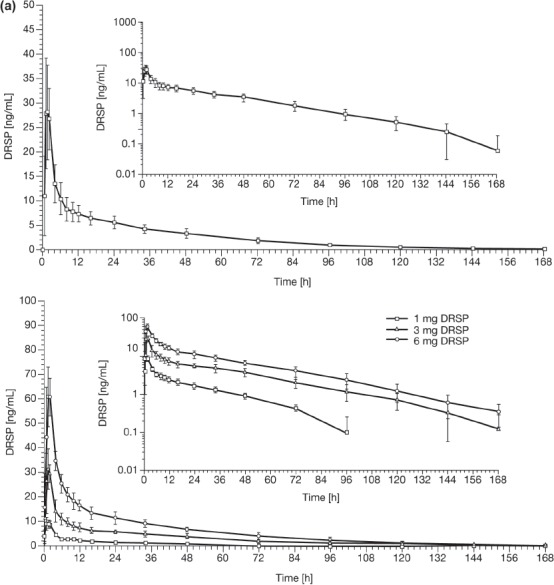

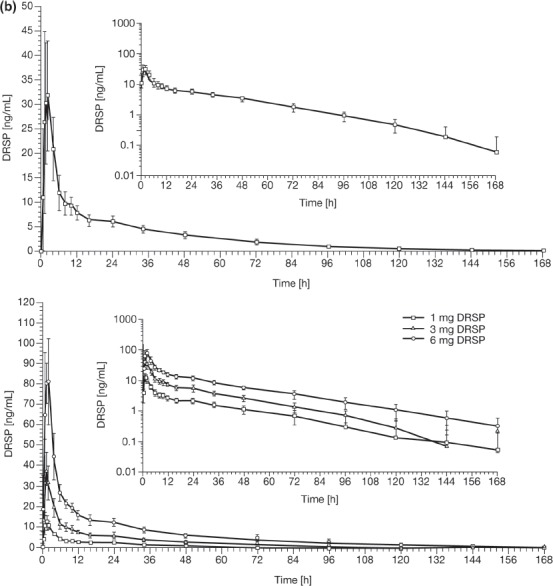
Drospirenone (DRSP) serum concentration versus time profile for DRSP after a single-dose administration of ethinylestradiol 0.02 mg/DRSP 3 mg and for DRSP after different single doses of DRSP (1, 3 and 6 mg) in (a) Caucasian women (Study 1) (logarithmic-scale shown inset) and in (b) Japanese women (Study 2) (logarithmic-scale shown inset). Results are presented as mean with standard deviation.

**Figure 2:**
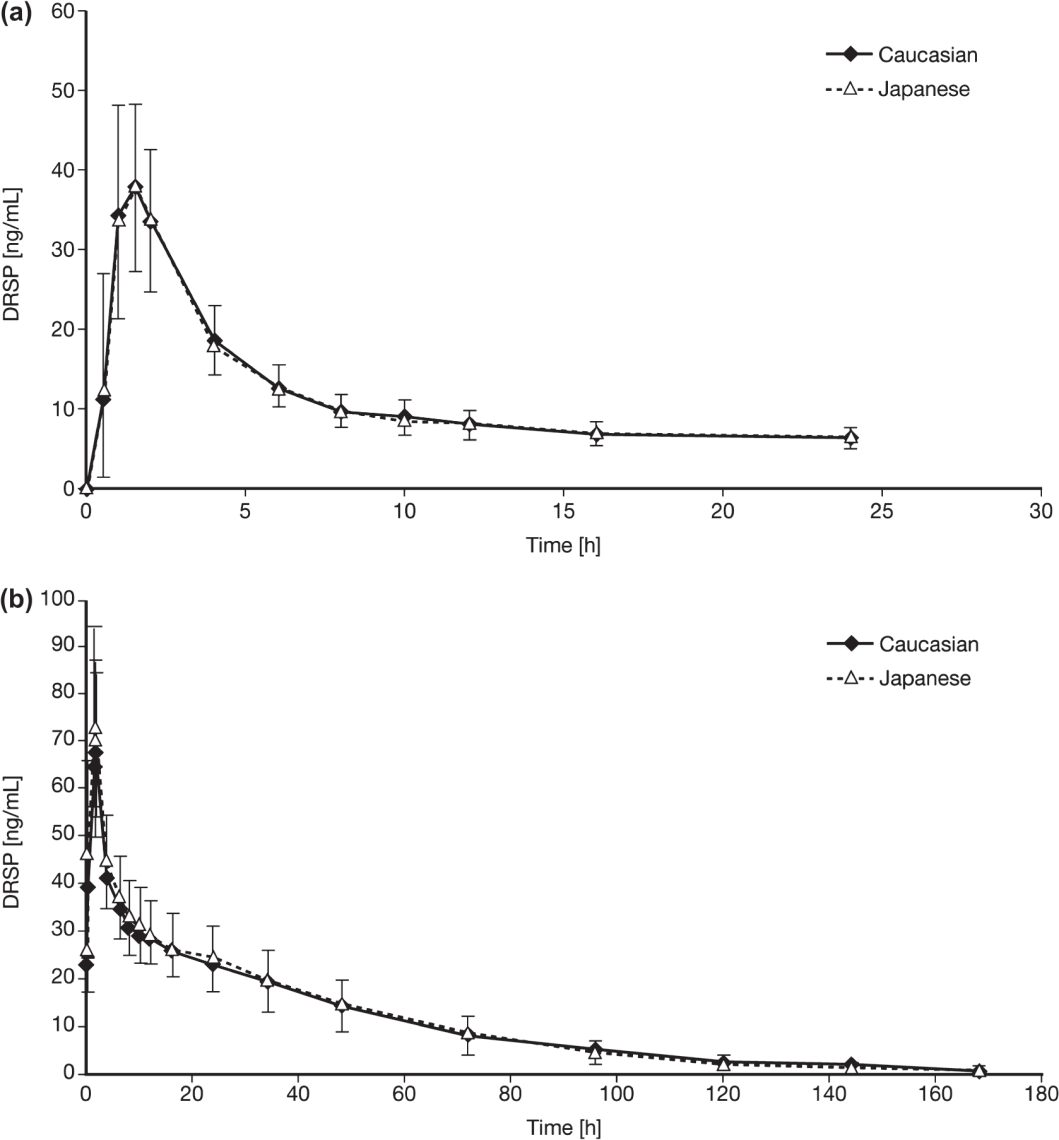
Drospirenone (DRSP) serum concentration versus time curves after (a) single (day 1) and (b) repeated (day 21) administration of an oral contraceptive containing ethinylestradiol 0.02 mg/DRSP 3 mg in healthy Caucasian (*n* = 23) and Japanese (*n* = 24) women (Study 3). Results are presented as mean + standard deviation (Japanese women) and mean − standard deviation (Caucasian women).

Figure 1 shows the serum concentration versus time profile for DRSP after single-dose administration of EE 0.02 mg/DRSP 3 mg and for single-dose DRSP (1, 3 and 6 mg) in Caucasian (Study 1) and Japanese (Study 2) women. In addition, DRSP serum concentration versus time curves on day 1 and after 21 days of daily consecutive administration of EE 0.02 mg/DRSP 3 mg (Study 3) in both ethnic groups are presented in Figure 2. The results of the DRSP pharmacokinetic analysis across the three studies are summarised in Table 2.

In the single-dose Studies 1 and 2, there was a linear dose-dependent increase in DRSP C_max_ and AUC across the range of doses used in both ethnic groups, whereas t_max_, t_½_ and oral clearance did not show dose-dependent changes. The co-administration of EE had no relevant effect on the pharmacokinetic parameters of DRSP when results of the 3 mg DRSP mono-therapy group were compared with those obtained after 0.02 mg EE/3 mg DRSP administration.

Following daily oral administration in Study 3, the mean t_max_ did not differ between the first and the last treatment days or between the two ethnic groups. Mean C_max_ on day 21 was approximately twofold greater than mean C_max_ measured on the first day of treatment. The geometric mean systemic exposure within a dosage interval (AUC_(0–24h)_) was 268 (CV = 19%) ng · h/mL and 271 (CV = 22%) ng · h/mL in Caucasian and Japanese women, respectively, after the first administration, and increased to 763 (CV= 17%) and 803 (CV = 24%) ng·h/mL, respectively, on the last day of administration. Therefore, steady-state systemic exposure was approximately three times higher after repeated doses than after a single dose, as reflected by the geometric mean accumulation ratios of 2.8 (CV = 20.3%) in Caucasian women and 3.0 (CV = 15.1%) in Japanese women.

Inter-ethnic comparison showed no differences in the primary target parameter, AUC_(0–24h)_ of DRSP at day 21 between young Caucasian and Japanese women; the ratio of the geometric means (Japanese/Caucasian) of the AUC_0–24h_ of DRSP on day 21 was 1.05 (90% CI, 0.95–1.17).

**Figure 3:**
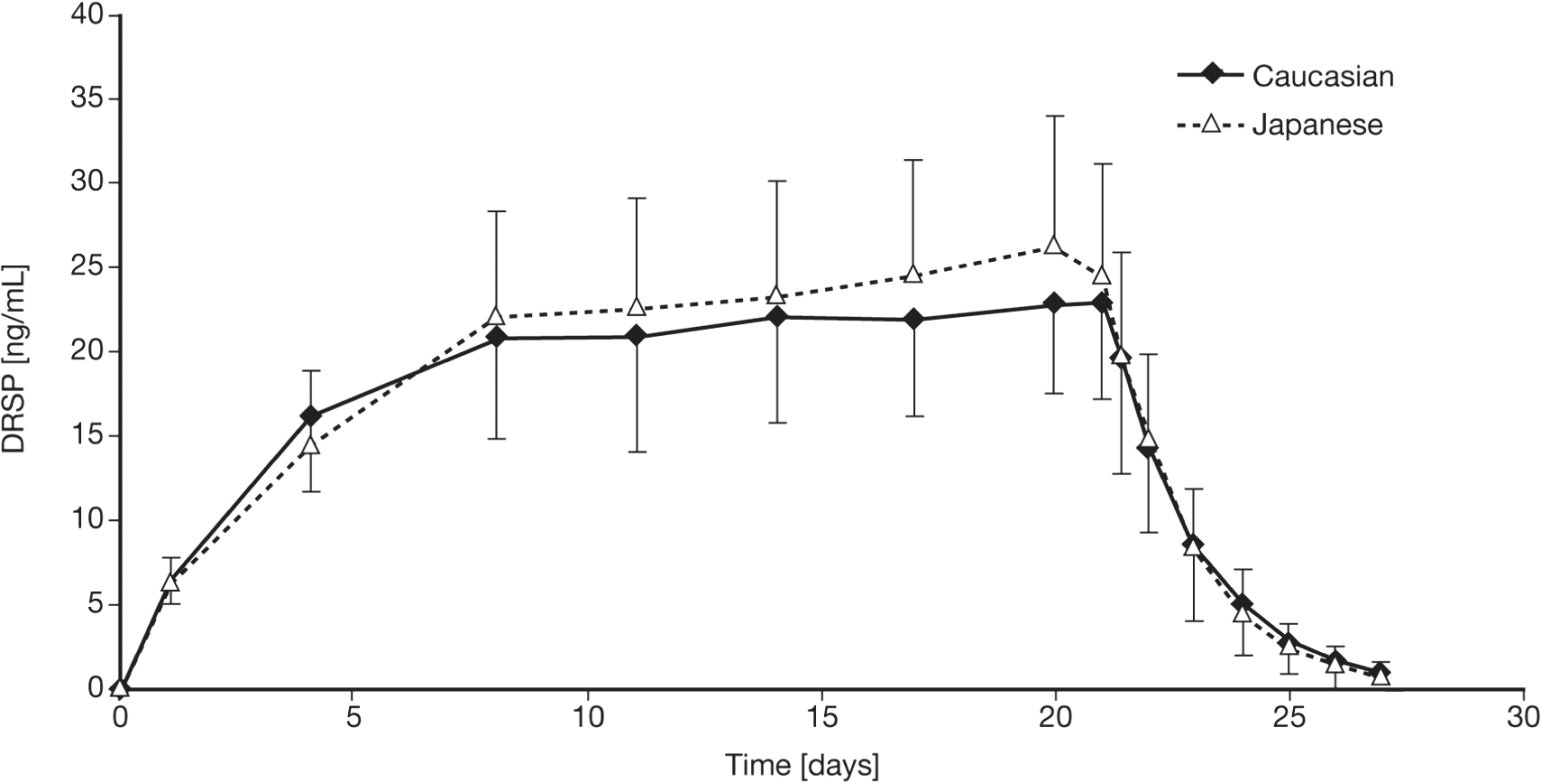
Mean serum drospirenone (DRSP) trough levels following daily administration of ethinylestradiol 0.02 mg/DRSP 3 mg over 21 days in young healthy Caucasian and Japanese women. Results are presented as mean + standard deviation (Japanese women) and mean − standard deviation (Caucasian women).

Mean serum DRSP trough levels following daily administration of EE 0.02 mg/DRSP 3 mg over 21 days are presented in Figure 3. Steady-state DRSP concentrations were achieved after approximately eight days of treatment in both ethnic groups. During the treatment-free period, although serum trough concentrations of DRSP declined during the seven days following the last tablet on day 21, mean concentrations of 0.96±1.01 ng/mL (Caucasian women) and 0.78±0.76 ng/mL (Japanese women) were measured by day 28, i.e., immediately before first tablet intake of the next treatment cycle.

**Table 2:** Pharmacokinetic parameters of drospirenone (DRSP) after different single doses of DRSP (1, 3 and 6 mg) (Studies 1 and 2) and after single administration (Studies 1–3) and repeated 21-day administration (Study 3) of ethinylestradiol (EE) 0.02 mg/DRSP 3 mg in healthy Caucasian and Japanese women. Results are presented as the geometric mean (geometric coefficient of variation), with the exception of t_max_ which is given as the median (range).

	*1 mg DRSP*		*3 mg DRSP*		*6 mg DRSP*		*EE 0.02 mg/DRSP 3 mg (single dose)*				*EE 0.02 mg/DRSP 3 mg (day 21)*	
					
*Parameter*	*Study 1 Caucasian (n − 6)*	*Study 2 Japanese (n − 6)*	*Study 1 Caucasian (n − 6)*	*Study 2 Japanese (n − 6)*	*Study 1 Caucasian (n − 6)*	*Study 2 Japanese (n − 6)*	*Study 1 Caucasian (n − 18)*	*Study 2 Japanese (n − 18)*	*Study 3 Caucasian (n − 23)*	*Study 3 Japanese (n − 24)*	*Study 3 Caucasian (n − 23)*	*Study 3 Japanese (n − 24)*
C_max_ (ng/mL)	9.85 (19%)	13.0 (29%)	33.9 (20%)	44.4 (21%)	62.5 (20%)	92.6 (13%)	30.9 (27%)	35.7 (31%)	38.4 (26%)	38.9 (32%)	70.3 (15%)	78.9 (23%)
t_max_ (h)	1.25 (1.0−2.0)	1.25 (1.0−1.5)	1.5 (1.0−2.0)	1.0 (1.0−1.5)	1.75 (1.5−2.0)	1.5 (1.0−2.0)	1.5 (1.0−4.0)	1.5 (0.5−4.0)	1.5 (1.0−2.0)	1.5 (1.0−2.0)	1.5 (1.0−2.0)	1.5 (1.0−2.0)
t_½_ (h)	25.5 (20%)	30.1 (34%)	28.5 (16%)	24.2 (24%)	26.3 (16%)	28.5 (21%)	27.7 (18%)	26.6 (19%)	ND	ND	30.8 (22%)	29.1 (18%)
AUC_(ng·h/mL)_	140 (9%)	182 (28%)	506 (22%)	458 (18%)	1007 (21%)	1051 (15%)	458 (18%)	494 (17%)	ND	ND	1811 (33%)	1852 (32%)
CL/F (mL/min)	119 (9%)	91.3 (28%)	98.7 (22%)	109 (18%)	99.3 (21%)	95.2 (15%)	109 (18%)	101 (17%)	ND	ND	ND	ND
AUC_(0−24h)_ (ng·h/mL)	ND	ND	ND	ND	ND	ND	ND	ND	268 (19%)	271 (22%)	763 (17%)	803 (24%)

AUC, area under the curve extrapolated to infinity; AUC_(0–24h)_, area under the curve up to 24 h after dosing; CL/F, total oral clearance; C_max_, maximum serum concentration; ND, not determined; t_½_, terminal half-life; t_max_, time to C_max_

### Pharmacokinetics of ethinylestradiol

**Figure 4:**
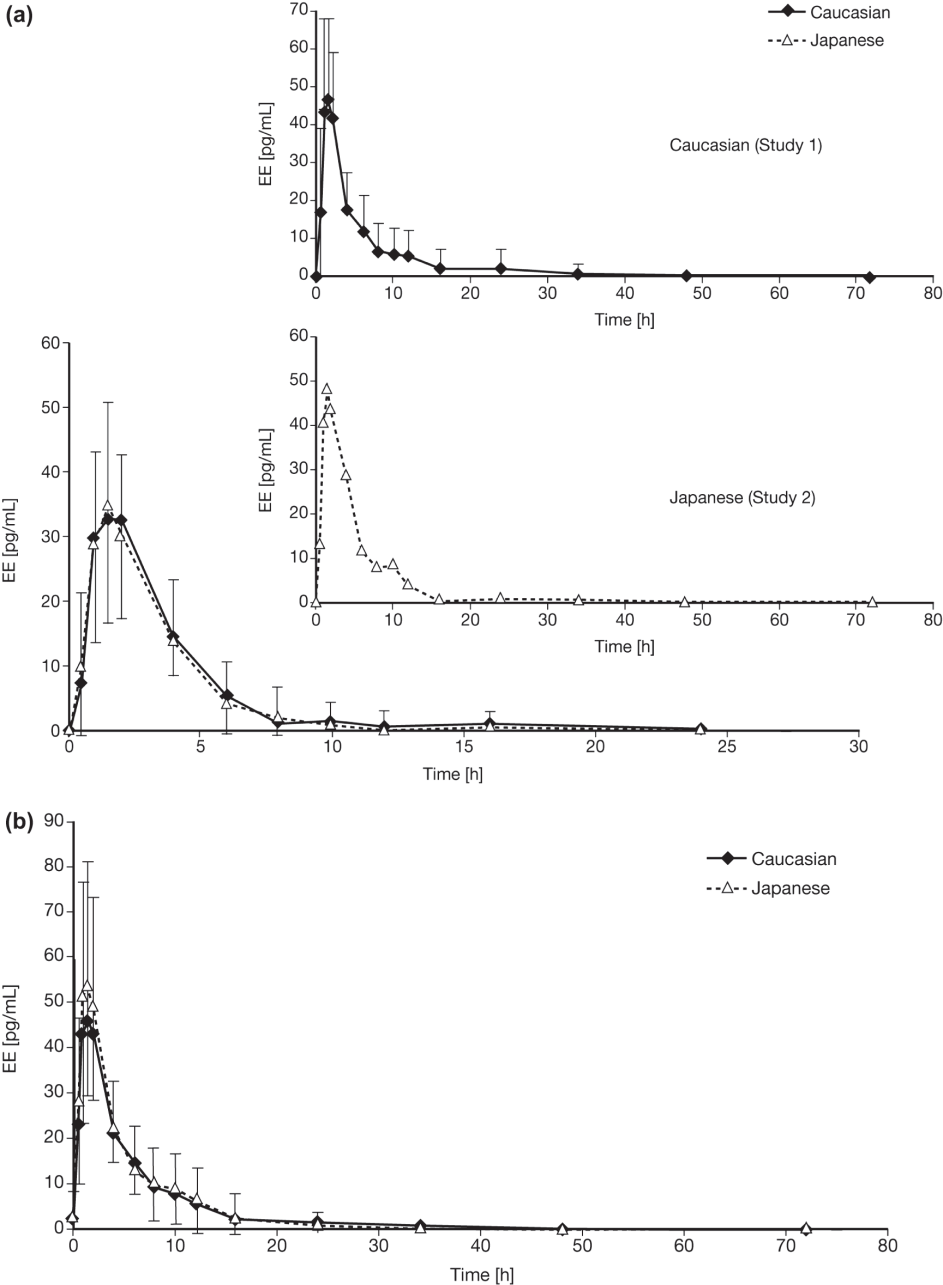
Ethinylestradiol (EE) serum concentration versus time curves after (a) single (day 1; inset Studies 1 and 2) and (b) repeated (day 21) administration of an oral contraceptive containing EE 0.02 mg/drospirenone (DRSP) 3 mg in healthy Caucasian (*n* = 23) and Japanese (*n* = 24) women (Study 3). Results are presented as mean + standard deviation (Japanese women) and mean − standard deviation (Caucasian women).

The EE serum concentration versus time curves following a single dose of EE 0.02 mg/DRSP 3 mg in Caucasian (Study 1) and Japanese (Study 2) women are presented as subfigures of Figure 4a. In addition, EE serum concentration versus time curves on day 1 and after 21 days of consecutive daily administration of EE 0.02 mg/DRSP 3 mg in both Caucasian and Japanese women (Study 3) are presented in Figure 4. The results of the EE pharmacokinetic analysis across the three studies are summarised in Table 3. The mean C_max_ had increased on the last day of treatment to about one and a half times the value on day 1. Based on mean AUC_(0–24h)_ values at day 1 and 21, the mean accumulation ratio for EE was 2.0 (CV = 45.6%) and 2.33 (CV = 40.8%) in Caucasian and Japanese women, respectively.

With regard to ethnic comparisons, there were no differences in EE AUC_(0–24h)_ between the two groups at day 21 (ratio of the geometric means; 1.02 [90% CI: 0.76 – 1.38]).

### Serum levels of sex hormone-binding globulin

**Figure 5:**
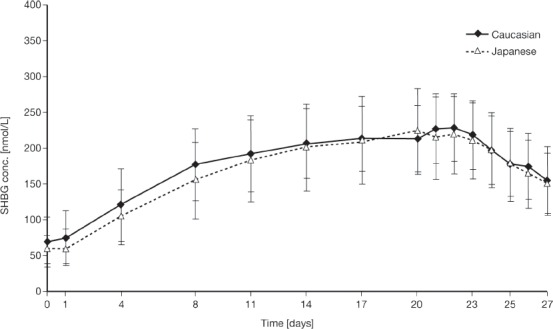
Mean sex hormone-binding globulin (SHBG) concentrations in serum after multiple dose administration of ethinylestradiol 0.02 mg/DRSP 3 mg in young healthy Caucasian and Japanese women over 21 days' administration. Results are presented as mean + standard deviation (Japanese women) and mean − standard deviation (Caucasian women).

Mean serum SHBG levels during EE 0.02 mg/DRSP 3 mg administration over 21 days in both Caucasian and Japanese women (Study 3) are presented in Figure 5. During the first week of EE 0.02 mg/DRSP 3 mg administration, the mean SHBG serum levels tended to be slightly higher in Caucasian than in Japanese women. Steady-state SHBG levels were attained by about day 15 of continuous daily administration of EE 0.02 mg/DRSP 3 mg. At the end of treatment, mean SHBG concentrations were 3.3-fold (Caucasian women) and 3.7-fold (Japanese women) higher than the respective mean pre-treatment concentrations, and declined within the seven days following treatment cessation to 2.3- and 2.5-fold pre-treatment concentrations in Caucasian and Japanese women, respectively.

### Safety

In Study 1, 16 AEs were observed in 14 out of 36 treated women (39%). Headache was the most common AE (six events: two women in the EE 0.02 mg/DRSP 3 mg group and three women in the DRSP 6 mg group). In Study 2, seven AEs were observed in seven out of 36 treated women (19%). These AEs were intermenstrual bleeding, headache, and upper respiratory tract infection. In Study 3, all women who entered the study had at least one AE. The most common drug-related AEs were headache (13 Caucasian women), intermenstrual bleeding (four Caucasian women, eight Japanese women), and dysmenorrhoea (five Caucasian women). Across all three studies, there were no serious AEs. All women experiencing AEs recovered completely.

## DISCUSSION

Overall, our observations suggest that ethnicity would have little or no impact on the pharmacokinetic profile of DRSP. There were no relevant differences between Caucasian and Japanese women in single-dose and steady-state pharmacokinetics of DRSP following administration of EE 0.02 mg/DRSP 3 mg, or following single oral doses of DRSP (1, 3 or 6 mg).

The current studies confirm previous observations^1^,^8^, that DRSP has a terminal half-life of about 30 hours and is, therefore, not completely eliminated during the hormone-free interval in a 21/7 cycle. Compared to a seven-day pill-free interval, the decline of DRSP levels during a four-day pill-free interval is expected to be less and can be estimated from the current steady-state data. The mean DRSP concentrations declined to 2.9 + 2.0 ng/mL in Caucasian and 2.5 ± 1.4 ng/mL in Japanese women by four days following treatment cessation, thus representing the expected mean concentrations at the beginning of the next treatment cycle in a 24/4 regimen.

The steady-state DRSP concentrations were reached by about day 8 in both Caucasian and Japanese women following daily administration of EE 0.02 mg/DRSP 3 mg. Both ethnic groups had very similar DRSP pharmacokinetic profiles at steady-state. The DRSP steady-state levels measured at day 21 would be maintained with continuous daily administration with EE 0.02 mg/DRSP 3 mg until day 24 or for as long as daily administration is continued.

The studies were designed to evaluate the influence of ethnicity on the pharmacokinetics of DRSP and EE and the number of women who completed each of the studies was sufficient for the ethnic comparison of the key pharmacokinetic parameters.

In general, the pharmacokinetic characteristics of DRSP and EE observed in our studies were consistent with those recorded in other studies in which combinations of DRSP and EE were orally administered (typically to healthy Caucasian women)^8^,^10^–^13^.

Because the aim of the studies reported here was to evaluate ethnic comparability of the pharmacokinetics of the active ingredients, these studies were not powered to any particular clinical endpoint. Clinical observations were reported with descriptive analyses but should be judged with caution because of the limited sample size.

**Table 3:** Pharmacokinetic parameters of ethinylestradiol (EE) after single administration (Studies 1–3) and repeated 21 day administration (Study 3) of EE 0.02 mg/DRSP 3 mg in healthy Caucasian and Japanese women. Results are presented as the geometric mean (geometric coefficient of variation), with the exception of t_max_ which is given as the median (range).

	*EE 0.02 mg/DRSP 3 mg (single dose)*				*EE 0.02 mg/DRSP 3 mg (day 21)*	
		
*Parameter*	*Study 1 Caucasian (n = 18)*	*Study 2 Japanese (n = 18)*	*Study 3 Caucasian (n = 23)*	*Study 3 Japanese (n = 24)*	*Study 3 Caucasian (n = 23)*	*Study 3 Japanese (n = 24)*
C_max_ (pg/mL)	46.1 (49%)	50.3 (42%)	32.8 (45%)	32.5 (52%)	45.1 (35%)	51.1 (53%)
t_max_ (h)	1.5 (1.0−4.0)	1.5 (1.0−4.0)	1.5 (1.0−2.2)	1.5 (1.0−2.0)	1.5 (1.0−2.0)	1.25 (1.0−2.0)
AUC_(0–24h)_ (pg · h/mL)	184 (77%)	205 (63%)	108 (52%)	96.5 (79%)	220 (57%)	225 (76%)
AUC_(0–last)_ (pg·h/mL)	162 (100%)	188 (75%)	ND	ND	206 (63%)	203 (84%)

AUC_(0–24h)_, area under the curve up to 24 h after dosing; AUC_(0–last)_, area under the curve up to the last data point; C_max_, maximum serum concentration; ND, not determined; t_max_, time to C_max_.

Although ethnic differences may cause variations in the characteristics of some drugs that could subsequently lead to differences in efficacy and safety between different populations, there are some properties of a drug that make it less likely to be affected by ethnic factors^14^. These properties include: linear pharmacokinetics; high bioavailability; minimal metabolism or metabolism via multiple pathways; or little potential for drug–drug, drug–diet and drug–disease interactions. We showed that DRSP has dose-linear pharmacokinetics across the doses used in the current study (1–6 mg), with dose-linear ity up to 10 mg demonstrated elsewhere^10^,^13^. In addition, DRSP has high bioavailability after oral administration (76%) that is unaffected by food intake^10^,^13^. Moreover, the metabolism of DRSP is only marginally catalysed by the cytochrome P450 system and, thus, is less likely to be affected by metabolic drug-drug interactions^15^. The co-administration of EE had no relevant effect on the pharmacokinetic parameters of DRSP based on the results from Studies 1 and 2.

There are large intra-individual and inter-individual variations in EE measurements^9^, as well as large intercentre and intra-centre differences^2^, which may, in part, contribute to the conflicting literature regarding the impact of ethnicity on the pharmacokinetic profile of EE. In our study, the pharmacokinetic profiles of EE following a single dose of EE 0.02 mg/DRSP 3 mg and at steady-state appear to be similar between the two groups.

The efficacy and safety of EE 0.02 mg/DRSP 3 mg has been extensively characterised in a predominantly Caucasian population. Although the use of EE in OCs is well established worldwide, DRSP has not been widely studied in other ethnic groups. The current studies provide information concerning the ethnic comparability of DRSP pharmacokinetics in general following administration of DRSP alone or in combination with EE. In addition, information on the steady-state pharmacokinetics of DRSP and EE after daily oral administration of 0.02 mg EE/3 mg DRSP was obtained.

Oral administration of DRSP (1–6 mg) or EE 0.02 mg/DRSP 3 mg produces systemic exposures to DRSP and EE in Japanese women that are similar to those in Caucasian women. Overall, ethnicity appears to have no clinically relevant impact on the pharmacokinetic profile of DRSP or EE in these two populations.

The results of the studies summarised here are relevant for the drug combination investigated. Extrapolation of these results to other progestogen/oestrogen combinations would require further justification. The sensitivity of a particular drug or drug combination towards ethnic differences and potential consequences with regard to dose adjustments should be investigated in dedicated studies.
